# Parametric Anatomical Modeling: a method for modeling the anatomical layout of neurons and their projections

**DOI:** 10.3389/fnana.2014.00091

**Published:** 2014-09-15

**Authors:** Martin Pyka, Sebastian Klatt, Sen Cheng

**Affiliations:** ^1^Department of Psychology, Mercator Research Group “Structure of Memory,” Ruhr-University BochumBochum, Germany; ^2^Faculty of Psychology, Ruhr-University BochumBochum, Germany; ^3^Faculty of Electrical Engineering and Information Technology, Ruhr-University BochumBochum, Germany

**Keywords:** 3d model, functional morphology, hippocampal formation, Blender, NEST, connection patterns, conduction latencies, brain anatomy

## Abstract

Computational models of neural networks can be based on a variety of different parameters. These parameters include, for example, the 3d shape of neuron layers, the neurons' spatial projection patterns, spiking dynamics and neurotransmitter systems. While many well-developed approaches are available to model, for example, the spiking dynamics, there is a lack of approaches for modeling the anatomical layout of neurons and their projections. We present a new method, called Parametric Anatomical Modeling (PAM), to fill this gap. PAM can be used to derive network connectivities and conduction delays from anatomical data, such as the position and shape of the neuronal layers and the dendritic and axonal projection patterns. Within the PAM framework, several mapping techniques between layers can account for a large variety of connection properties between pre- and post-synaptic neuron layers. PAM is implemented as a Python tool and integrated in the 3d modeling software Blender. We demonstrate on a 3d model of the hippocampal formation how PAM can help reveal complex properties of the synaptic connectivity and conduction delays, properties that might be relevant to uncover the function of the hippocampus. Based on these analyses, two experimentally testable predictions arose: (i) the number of neurons and the spread of connections is heterogeneously distributed across the main anatomical axes, (ii) the distribution of connection lengths in CA3-CA1 differ qualitatively from those between DG-CA3 and CA3-CA3. Models created by PAM can also serve as an educational tool to visualize the 3d connectivity of brain regions. The low-dimensional, but yet biologically plausible, parameter space renders PAM suitable to analyse allometric and evolutionary factors in networks and to model the complexity of real networks with comparatively little effort.

## Introduction

Computational simulations of neural networks have become an important tool to untangle the relationship between the function of a network and its structural properties. There are several levels on which artificial neural network can capture properties of the biological ideal. At the neuronal level, these are, for example, the spiking dynamics (Dayan and Aboot, 2001), dendritic morphology (London and Häusser, 2005; Cuntz et al., 2010), and the rules underlying structural (Butz and van Ooyen, 2013) and spike-timing dependent plasticity (Morrison et al., 2008). At the network level, connections between neurons and their spatial distances are of particular importance. They can have an influence on conduction delays, which in turn can be functionally important (Carr and Konishi, 1988; Blumberg, 1989; Bartos et al., 2002; Maex and De Schutter, 2003; Soleng et al., 2003; Gong and van Leeuwen, 2007; Buzsáki, 2010; Hu et al., 2012).

Temporal dynamics of neural activity and plasticity rules can be mathematically described with comparatively great accuracy and they can be efficiently translated into a programming language. Several well-established tools, like Neuron (Hines and Carnevale, 2001), GENESIS (Beeman, 2005), NEST (Eppler et al., 2008), and Brian (Goodman and Brette, 2008) can be used for this purpose. In order to integrate spatial properties into the network, e.g., location dependent connections and conduction latencies, specialized tools have been developed. For instance, in Neuroconstruct (Gleeson et al., 2007), neurons with realistic morphologies or abstract probability distributions can be imported or generated. They can be either manually placed in space or distributed based on user-defined functions or across simple geometric shapes (e.g., a cube). Recently, a new tool called NeuralSyns (Sousa and Aguiar, 2014) was presented, which allows the processing of up to 10^7^ synapses and the real-time visualization of spiking activity and connections. Neurons can be placed in space and connected with each other using procedural approaches. For simulations, in which the topographical arrangement of neurons is of predominant importance, the topology toolbox for NEST (Eppler et al., 2008) and Topographica (Bednar, 2009) provide helpful tools to set up 2d sheets of neurons and to connect them with each other using pre-defined kernel functions.

**Figure 1:**
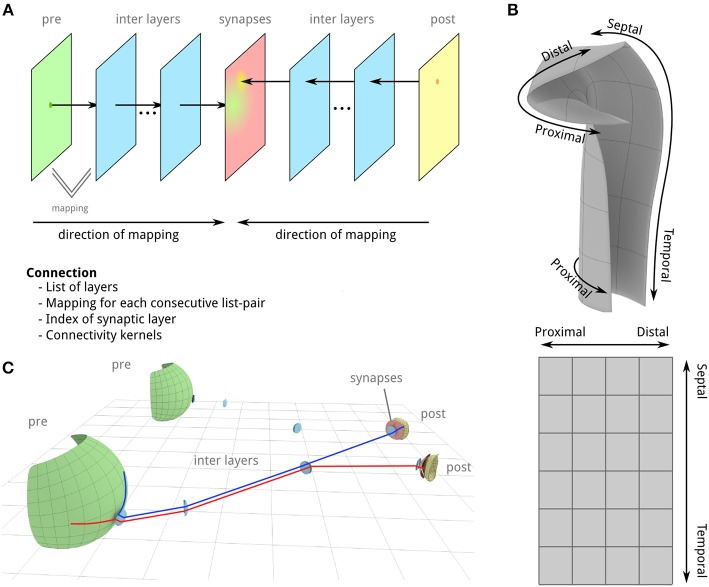
**Illustration of basic concepts in PAM. (A)** *2d* layers define the location of neurons, their projection directions and which neurons form synapses. Probability functions for pre- and post-synaptic neurons are applied on the surface of the synaptic layer to determine connections between the two neuron groups. **(B)** A layer is defined as a 2d manifold (a deformed 2d surface) in 3d Euclidean space (upper part). Each point on the surface is therefore described by x, y, and z coordinates. The relative positions on the flattened surface can be described in uv-coordinates (lower part) which may correspond to anatomical axes. This example depicts a rough sketch of CA1-3. **(C)** A simplified example of the outlined idea for the visual pathway from the retina (green surface) to the lateral geniculate nucleus (LGN, yellow) (proportions do not match). Different mapping techniques (see chapter “Mapping”) allow a location-dependent mapping of a neuron position in the pre-layer onto the synaptic layer (red layer) of the left and right LGN, respectively.

These tools have proven to be of great value in models of local regions in the brain and of connection principles that do not rely on the anatomy of biological brain regions (Gouwens and Wilson, 2009; Rothman et al., 2009; Bednar, 2012; Azizi et al., 2013; Helias et al., 2013; Mattioni and Le Novère, 2013; Stevens et al., 2013). However, they barely support the integration of large-scale anatomical properties obtained from histological and imaging data or tracer studies. In fact, converting anatomical knowledge to a formal description of connections and conduction latencies between a large number of neurons poses to be a very hard problem, as axonal and dendritic projections and the location and orientation of neurons follow complex non-linear patterns. This problem is, for example, very apparent in the hippocampal formation. Besides global axes (such as anterior-posterior, dorsal-ventral) along which the shape of the different layers of the hippocampus can be described, projection patterns within these layers (e.g., CA3, CA1) follow local axes (e.g., proximal-distal, septal-temporal) (Andersen et al., 2006). The orientation of these local axes, however, depends on the shape of the hippocampus along the global axes (e.g., Figures 1C, **5C**). Topological relations between CA1 and entorhinal layers remain roughly preserved. However, connection distances between those layers vary widely as different parts of CA1 and entorhinal cortex have different non-linear tracks and therefore varying distances to each other (Van Strien et al., 2009).

This dependency between the shape, location and projection pathways of neural layers on the one hand and connections and path lengths on the other, can be basically found in the entire brain. The visual pathways are a good example for topological mapping between two distant layers that differ in their anatomical shape (Rodieck, 1979). Cortical layers connect to their immediate neighbors but also to more distant regions via long myelinated axons forming the white matter (Passingham and Wise, 2012).

More and more detailed knowledge about biological neural networks becomes available through numerous independent studies and large initiatives like the Human Brain Project (Markram, 2012) or the data portal of the Allen Brain Atlas (Jones et al., 2009). By contrast, currently available tools for creating 3d neural networks do not provide the possibility to efficiently make use of the vast amount of data that are publicly available.

With Parametric Anatomical Modeling (PAM), we propose a technique and a Python implementation to close this gap. The basic idea of PAM is to trace neural, synaptic and intermediate layers from anatomical data and relate those layers to each other. With a set of mapping techniques, complex relationships between those layers can be defined to determine how axonal and dendritic projections traverse through space and where synapses are formed. A powerful feature of PAM is that spatial relations between and within layers can be combined to derive connections and distances between neurons. Furthermore, two- and three-dimensional experimental data (e.g., gene expression maps, marked neurons) can be integrated in the model to describe neural density or functional properties of neurons. As a side effect, neural networks created using PAM are of high educational value as the depiction of neural layers created from anatomical data along with the selective visualization of axons, dendrites and synapses can be explored in 3d and clearly demonstrate how the layers are wired.

In the following, we first introduce the principles that PAM is built on and then an implementation of PAM in the 3d software Blender. Subsequently, we apply PAM to build a model of the hippocampal formation in the rat. Finally, we find that these models can lead to new insights about brain structures and, potentially, functions.

## The model

### The basic concepts

The most important concept in PAM is the “layer” (Figure 1). A layer is a two-dimensional grid-like structure that can be deformed in 3d space to resemble any anatomical layer in the brain. Layers are the structures that can be directly created from anatomical data to denote, for instance, the location of pre- and post-synaptic neurons, synaptic layers (SLs) and intermediate layers that help to define the trajectories of axons and dendrites. Using a simple set of mapping techniques (see below), various relations between layers can be described in order to create location-dependent trajectories of neurons in 3d space. These trajectories are used to determine connections and distances between neurons which may affect the transmission delay. As will become apparent in the following, when we use PAM to implement a model of the hippocampal formation, complex connection patterns between layers can be expressed easily.

Note, that the wiring of the network is defined solely on the level of layers and not for single neurons. This approach corresponds to the notion that in real networks 3d patterns define where in space precursor cells proliferate and in which directions axon and dendrite cones grow. PAM uses these low-dimensional but biological plausible categories to define the architecture of neural networks. Groups of identical neurons are then distributed over the layer with a given density. Their connections to other neurons is a result of their relative location to other neurons and their projection direction across intermediate and SLs. PAM does not include a developmental component such as structural development through gene regulatory networks or cell migration. Instead, our approach rather aims to understand the functional implications of the developed structure.

A network is defined by mappings between pre-synaptic and a post-synaptic neural layer (green and yellow layer in Figures 1A,C) on SL (red layer in Figures 1A,C). A layer can be involved in an arbitrary number of mappings and the same layer can be both the pre- and post-SL of a mapping to account for recurrent connectivity. Thereby, projections of different neuron groups located on the same layer to different regions in the network can be described. The definition of relations between layers is a general form that describes how dendrite and axons traverse through layers until they form synapses. With these definitions, the corresponding position on the intermediate and SL can be computed for any point on a pre- or post-SL.

Connections between pre- and post-synaptic neurons are determined by probability functions that define for any relative position on the SL its probability for generating synapses (Figure 1A, red layer). Using probability functions reflects the assumption that all neurons on a layer that belong to the same neuron type have the same genetic code and emerged through cell proliferation. The individual morphological structure of each neuron is an instance of a general connectivity pattern that the neuron encodes influenced by other factors, such as structural and synaptic plasticity. The probability functions represent the general connectivity pattern.

A special feature of layers in PAM is that any point on the layer can be described by its xyz-coordinates in Euclidean space and in surface-coordinates, commonly called uv-coordinates in 3d graphics. uv-coordinates are generated when the 3d mesh of a layer is unfolded until all points of the layer are mapped onto a 2d plane (Figure 1C). As we will see in the next chapters, this transformation can be used to determine distances and connections on the surface level. Moreover, it allows to describe anatomical properties either along the spatial axes (xyz) or along anatomical axes (uv) which are not necessarily straight (like the proximal-distal axis in the hippocampal formation).

### Mapping

**Figure 2:**
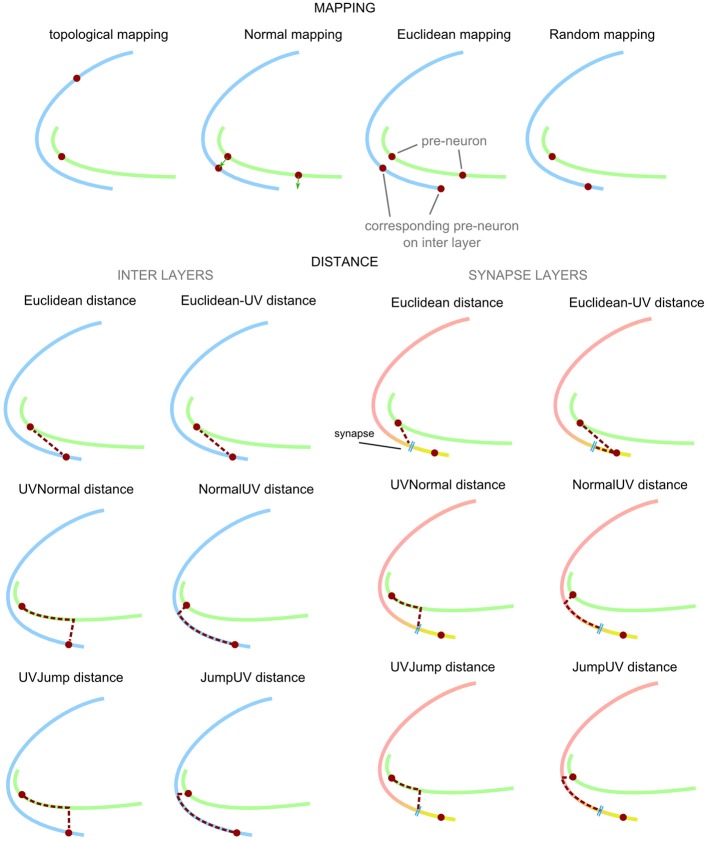
**List of mappings between layers and techniques to compute distances.** In order to create axonal and dendritic connections in 3d space, neuron positions are mapped between layers. When the internal mesh-structure between layers is identical, neurons can be directly mapped using topological mapping. Otherwise, normal-, Euclidean-, and random-based are available. The computation of connection length can combine spatial relations between layers and within layers. On an interlayer, connection length is computed based on the trajectory to the mapped neuron on the next layer. On the synaptic layer, the pre- and post-synaptic trajectory ends at the synapse. Euclidean mapping always computes the shortest distance to the mapped neuron or synapse. Normal mapping computes the distance based on the normal of the surface at the neuron position. Jump mapping computes the smallest distance to the next layer. Techniques with “UV” as affix add spatial distances along the current layer to the distance.

A central feature of PAM is that through various mapping techniques spatial relations on the surface of layers and spatial distances between layers can be combined to compute connections and distances between neurons. The top row in Figure 2 depicts the four types of mappings between two layers. In the following, we explain each mapping in more detail and outline its use cases.

#### Topological mapping

When two layers have the same internal topology (e.g., identical number and ordering of vertices and definition of quads and triangles), for any point on the first layer its corresponding position on the second layer can be directly computed. This mapping technique is useful whenever topological relations between neurons should be preserved independent from the origin and target location of axons and dendrites in space. The most obvious example for this is the mapping between photoreceptor cells in the retina to V1, where intermediate layers could be used to layout the realistic trajectory of the fibers to the visual cortex. But also the mapping of the dentate gyrus layer on a SL around CA3 in the hippocampus could make use of topological similarities to constrain the axonal projections along the septo-temporal axis.

#### Normal mapping

Any point *p* on a layer *X* is mapped on another layer *Y* by computing the intersection between the line normal to *X* through the point *p* and layer *Y*. If there is no intersection, there is no connection. This mapping technique can be used when the projection direction of neurons solely depends on the layer it is located in (e.g., cortical layers). Furthermore, this mapping technique can be helpful to selectively map subareas of a layer onto certain target regions (e.g., connections from the lateral and medial entorhinal cortex to different parts of the dentate gyrus, see exemplary demonstration section).

#### Euclidean mapping

Euclidean mapping computes for a given point *p* on the first layer the closest point on the next layer. Such a mapping can be useful, when the relative position of neurons on the first layer and its proximity to the target layer determine their entry direction on the target layer. This can be helpful if the curvature of layers in space do not allow a reliable mapping between layers based on normal mapping.

#### Random mapping

The random mapping maps a point *p* on one layer to an arbitrary location on the next layer. This mapping is useful when the projection kernels of neurons are well-defined while the axonal or dendritic projections through space are randomly distributed across brains.

#### Distance calculation

The connection distance between a pre- and a post-synaptic neuron along the axon and dendrite is an important piece of information, for instance, when conduction latencies should be part of the network simulation. PAM includes several methods to measure the distance between a neuron and a synapse incorporating spatial distances on a layer and between layers (Figure 2). UV-distances are needed when neurites grow along a certain layer that is curved in 3d space. The projections of pyramidal cells in CA3, for example, traverse the stratum oriens and stratum radiatum in CA3 and CA1, which has a strong effect on the overall pathlength between pre- and post-synaptic neurons (Andersen et al., 2006). A similar effect can also be found in the projections of pyramidal cells in the cortical layers V and VI (Passingham and Wise, 2012).

Euclidean distances between two layers correspond to connections along the shortest paths in space. These can be connections through the whole nervous system, like thalamo-cortical connections or sensory pathways but also more direct connections between cortical layers.

Note that the distances computed by any of these methods represent estimates of the lower bounds since the convoluted morphology of real dendrites and axons may result in longer pathways and therefore longer latencies between two endpoints.

Electrophysiological studies have shown some variability in the conduction latency per mm (Ferster and Lindström, 1983; Swadlow, 1994; Soleng et al., 2003). The assumption in PAM is that this variability emerges as a result of variability in the development that may lead to, e.g., different neurite lengths, different degrees of myelination, etc. To account for this variability, the conversion from connection length to conduction latency in PAM introduces a certain degree of variance based on experimental data.

### Connectivity kernels

**Figure 3:**
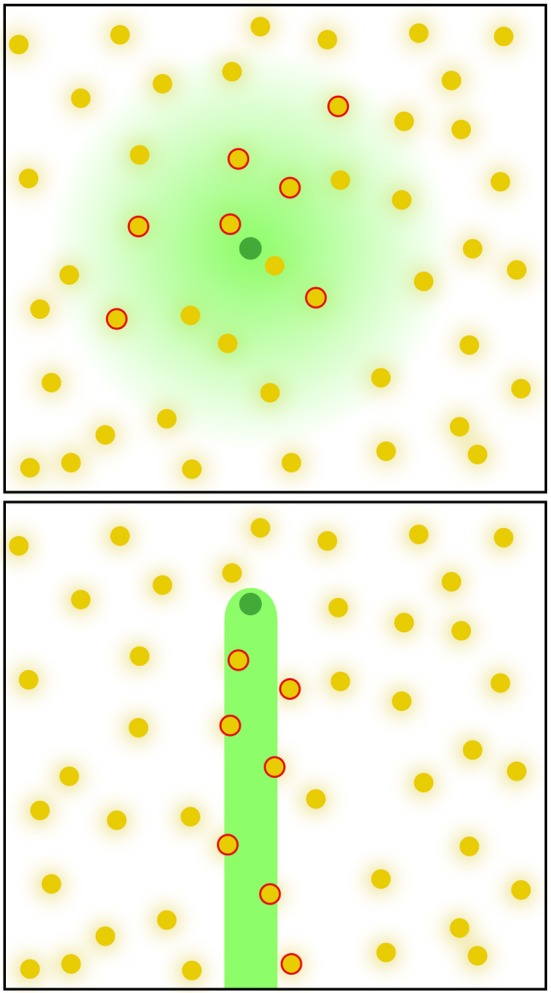
**Two examples for connectivity kernels.** Arbitrary connectivity kernels can be defined to generate synapses between pre- and post-synaptic neurons. Kernel functions are mapped onto the synaptic layer and define the probability for a neuron to form synapses at a relative position in the synaptic layer. Illustrated are two different kernel functions (green shading) for two pre-synaptic neurons (green dots) and the potential post-synaptic partners (yellow dots), which have their connectivity kernels (not shown). The joint probability of pre- and post-synaptic kernels determines if and where a synapse if formed.

Synaptic connections have to follow functional as well as anatomical constraints. Synapses have a physical location in space and, more often than not, pre-synaptic neurons connect preferentially to post-synaptic neurons in certain locations (Passingham and Wise, 2012). To model both the connectivity preference and the spatial distribution of synaptic connections, we employ the following method. Pre- and post-synaptic neurons are assigned spatial locations in the SL in uv-coordinates, *^z^*pre and ^*z*^post, respectively. This position is somewhat arbitrary and becomes meaningful only together with the connectivity kernel *p* (*z*|*z*_neuron_) that determines the probability of a neuron forming synapses in location *z* (Figure 3). Roughly speaking, the kernel models the reach of the dendritic tree or the axon, and the density with which synapses are formed. An arbitrary number of parameters can be integrated in the kernels to further parameterize it. In general, the shape of the kernel might depend on the position of the neuron x, y, z and/or uv-coordinates and can be defined by the user. PAM currently includes a few connectivity kernels, such as a 2d-Gaussian distribution, or a 1d-Gaussian distribution along a local anatomical axis. The user can easily add new kernel functions (e.g., a power law distribution) by creating python module in the kernel folder (see gaussian.py in the code for a template).

To make the problem of determining synaptic connections more tractable, we assume that the probability of having a synaptic connection is the product of the pre- and post-synaptic connectivity kernels.

(1) $$p\left(z|z_{\text{pre}},z_{post}\right)=p\left(z|z_{\text{pre}}\right)p\left(z|z_{\text{post}}\right)$$

The task of finding synapses is equivalent to sampling from this distribution, which is simple to implement.

The general form of the function also allows us to define connectivity kernels in which the position of the neuron on the surface influences the shape of the kernel. Thereby, anatomical axes (e.g., the proximal-distal axes in the hippocampal formation) can be integrated in the definition of the kernel (see Discussion for more details).

However, in a network with realistic numbers of neurons and synapses, the computation of synaptic connections can be very time consuming. If every potential connection between *n* pre-synaptic and *m* post-synaptic neurons is evaluated at *c* spatial locations, the computational effort scales as *O(cnm)*, a large number even in a small rat brain. In some cases where modeling the connectivity precisely is important, there might be no other alternative. If, on the other hand, the details are less important than the gross features of the connectivity, we can use an approximate sampling algorithm that provides a trade-off between mathematical accuracy and computational efficiency.

If synaptic connections are formed sparsely, we can save computational time by systematically skipping partners that have a very low connection probability. The specific algorithm is as follows.

**Figure 4:**
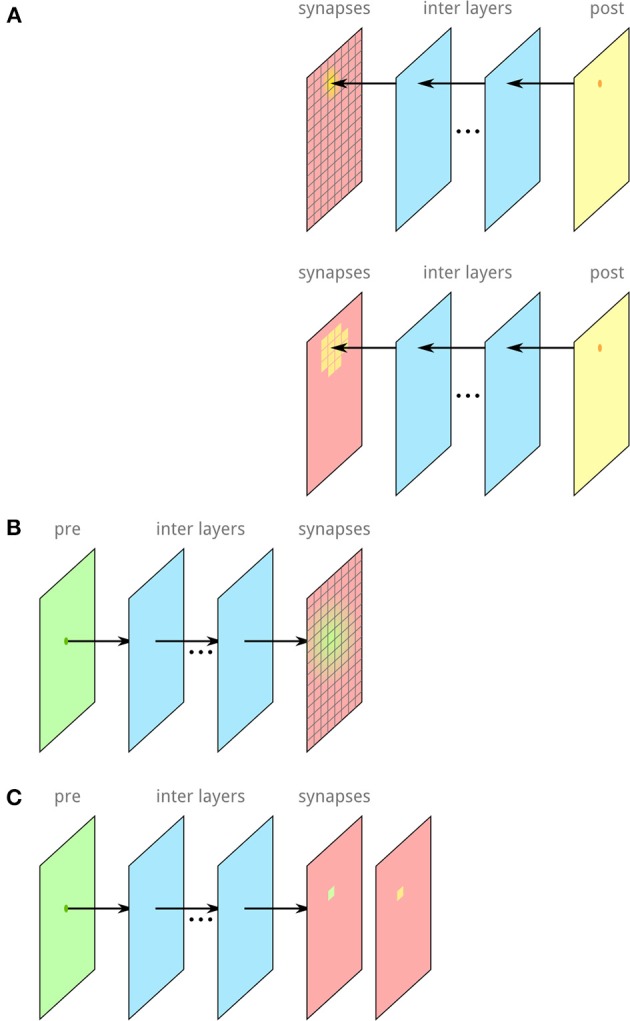
**Illustration of the algorithm to accelerate the computation of synapses and connections between neurons. (A)** The synaptic layer is divided into a raster of *c* bins. Each post-synaptic neuron is mapped on the synaptic layer and assigned to each bin of the synaptic layer with its probability for synapse-formation in this particular bin. **(B)** Each pre-synaptic neuron is mapped on the synaptic layer. For the number of synapses, we want to generate, bins are samples following the connectivity kernel of the pre-synaptic side. **(C)** In each selected bin, a post-synaptic neuron is randomly selected, incorporating the probabilities for the post-synaptic neurons.

Step 1: The SL is divided into *c* bins (Figure 4), where *c* is chosen appropriately depending on the number of neurons and the size of the connectivity kernels.
Step 2: Each post-synaptic neuron is mapped onto the SL and added to every bin *z_i_* in which the connectivity kernel exceeds a certain threshold, i.e., *p* (*z_i_*|*z*_post_) ≥ *p*_0_ (see Section Methods). The values *p* (*z_i_*|*z*_post_) are stored with the neuron id in the bin for later use.
Step 3: Each pre-synaptic neuron is mapped on the SL and we sample as many times from its connectivity kernel *p* (*z*|*z*_pre_) as we need to generate synapses for this pre-synaptic neuron (Figure 4B). Each sample yields a bin in the SL *z_j_*, in which the pre-synaptic neuron forms a connection. This sampling can be further sped up by skipping low-probability bins, for which *p* (*z*|*z*_pre_)≤ *p*_1_.
Step 4: From each bin in the previous step, we determine the post-synaptic neuron to connect by sampling from *p* (*z*_post_|*z_j_*). These probabilities are related to the probabilities stored in Step 2 through Bayes' theorem
  (2) $$p\left(z_{\text{post}}|z_{j}\right)=p\left(z_{j}|z_{\text{post}}\right)\frac{p\left(z_{\text{post}}\right)}{p\left(z_{j}\right)}$$
Since *p* (*z*_post_) is the same for every post-synaptic neuron *p* (*z*_post_|*z_j_*)∝ *p* (*z_j_*|*z*_post_).

The computational costs for this algorithm scales with the number of synapses *s* = α*nm*, which is significantly better than the exact algorithm because for large networks ^α^ is generally a small value.

## Methods

### Implementation of the framework

**Figure 5:**
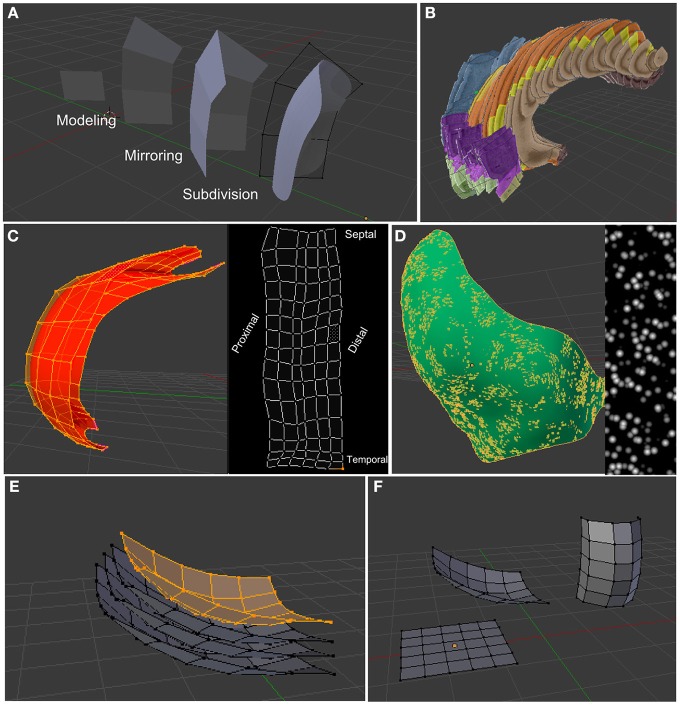
**Some functions of Blender that are important for PAMs. (A)** Various modeling techniques and non-distructive modifiers (like the Mirror- or Subdivision-modifier) allow an efficient creation of *3d* models of anatomical regions. **(B)** Anatomical slices along with transparency values can be displayed for easier *3d* tracking of neural layers (here the Hippocampus Brain Atlas). **(C)** *3d* objects can be unwrapped on a *2d* plane to assign non-linear anatomical axes to them. **(D)** Textures can be used to define the probability distribution of neurons or synapses along xyz- or uv-axes. **(E)** Using the shrink-fatten operator, layers can be easily generated from existing layers. **(F)** Duplicates of layers make it easy to map locations of one layer on other layers, as their internal ordering of vertices and edges is the same.

PAM is a general approach to generate artificial neural networks based on anatomical data. To apply this technique, tools are needed to model and define the relationships between the layers. Therefore, we developed the functionality for defining parametric anatomical models (PAMs) in the open source 3d software Blender^1^. Using an existing 3d software for creating PAMs has the advantage that most of the tools for creating 3d layers are already implemented. Figure 5 lists some of the functions that are of particular relevance for creating PAMs and that are generally implemented in most 3d tools. Most importantly, duplication of layers make it easy to map points between layers with arbitrary, but identical, shapes (Figure 5F). Furthermore, important for PAM is that 3d shapes can be unfolded to assign non-linear axes to the object (Figure 5C). The development of neuroscientific tools, such as Py3DN (Aguiar et al., 2013) and BrainBlend (Pyka et al., 2009), and tools for other disciplines, such as BioBlender (Andrei et al., 2012) and MORSE ^2^ as Blender add-ons, suggest that Blender could become a unifying Python-based platform for developing scientific tools.

There were additional reasons for implementing PAM in the Blender environment. Because of its strong support for Python and its open application programming interface (API), Blender can be used as an integrated development environment for creating new tools and amending existing tools such as NEST. PAM for Blender consists of a set of add-ons and Python modules that extend the functionality of Blender to generate and relate anatomical layers to each other and to create neural networks for the networks simulator NEST. These tools along with example files and video tutorials are freely available ^3^. In the following, we give a short introduction into the available tools by explaining the workflow for creating PAMs.

#### Creating anatomical layers

First, layers need to be created that define the location for the cell bodies of neurons and for their synapses. Depending on the brain region, intermediate layers might be included to describe important landmarks for the trajectories of neurites. Since 2d and 3d images can be imported into Blender, atlas data or anatomical data, such as histological images or 3d data acquired through computer tomography or magnetic resonance imaging, can be used to support the modeling process. The depiction of metric units within the modeling environment allows to model the 3d structures with the correct scaling. All layers can be automatically unfolded to make uv-coordinates for the layers available. This part relies on Blender's internal tools and requires some modeling skills. However, once a brain region has been modeled as layers, it can serve as a template for a variety of neural network models.

#### Setting neural parameters

**Figure 6:**
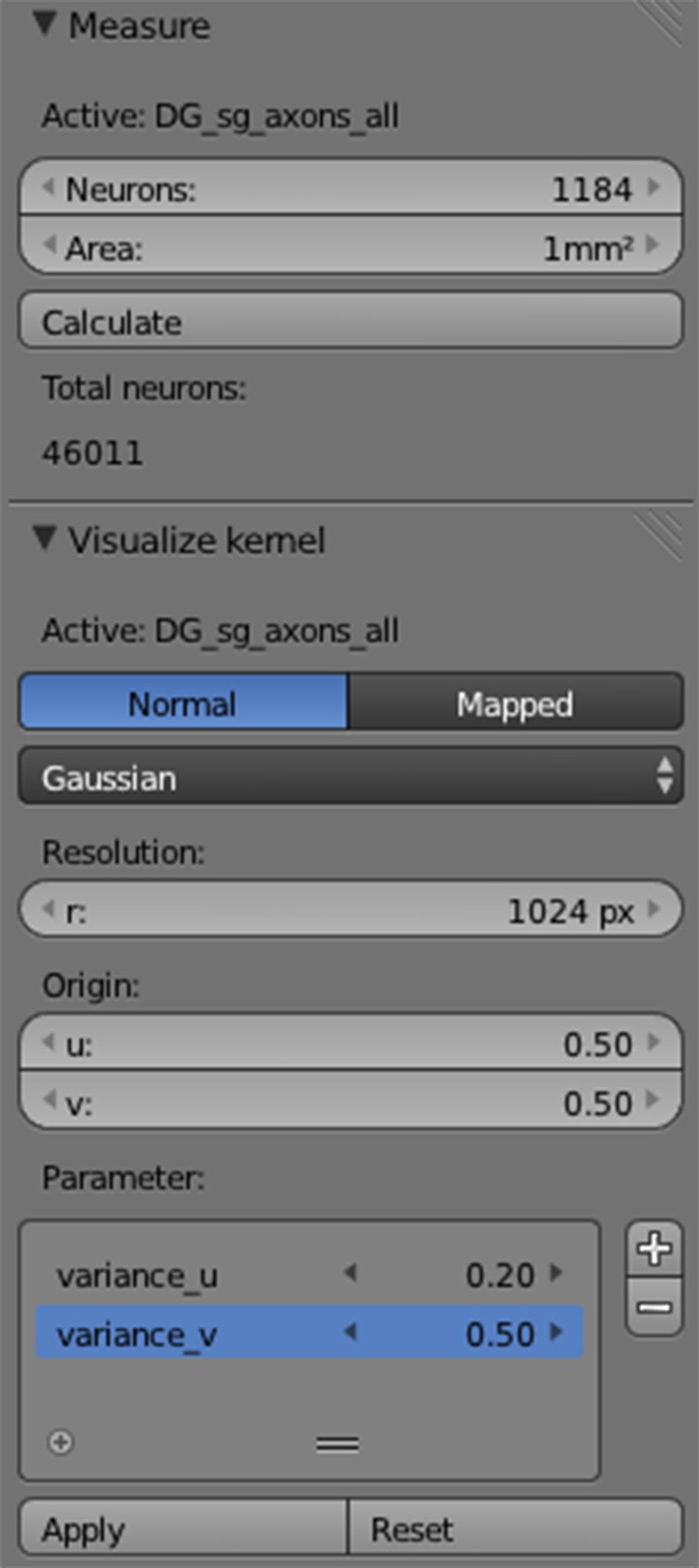
**PAM add-on for Blender.** A user-interface for computing the surface area, neuron numbers, and connection kernels for reconstructed layers.

The traced anatomical layers already allow first inferences. For example, the user can obtain the surface area of the layers and calculate the total number of neurons hosted by the layer, given for example the neural density per mm^2^. For each neuron group in a layer, the number of neurons that should be used in the simulation, can be defined. The PAM add-on for Blender provides a user interface for calculating the surface area, number of neurons and for visualizing the connectivity kernels on the SL (Figure 6). However, everything can also be set up using Python scripts. The cell bodies of the neurons are usually homogenously distributed over the surface. Additionally, using build-in functions of Blender, 2d and 3d textures (like gene expression maps or gene marker data) can be mapped on the surface of the layers to determine the location-dependent density of neurons.

#### Creating mappings

Each layer can host several neuron types which in turn can connect to several regions. Each mapping is defined by

- a set of layers (pre-, post- and synaptic layer; and optionally intermediate layers)
- the neuron types which are connected
- the mapping between successive layers
- the way distances between successive layers should be calculated
- the connectivity kernels for pre- and post-synaptic neurons
- the number of outgoing (or incoming) connections

Note, that a 3d-layer can have multiple roles in the definition of a mapping. For example, it can be pre- and post-SL, and technically even the SL at the same time. Therefore, recurrent connections can be described using the same syntax as feedforward connections. We provide PAM modules for defining connections and computing the mapping, the synapses between neurons and their connection lengths. Furthermore, neurons and connections can be visualized to obtain a qualitative impression of the setup and to manually adjust the connectivity kernels. Several video tutorials and a wiki on the project website document how connections in PAM can be defined to rebuild connectivity patterns of real neural networks (http://cns.mrg1.rub.de/index.php/software).

#### Export connectivities and distances

Connections and distances between neurons can be exported as CSV-file or as Python pickle-file for further processing in an arbitrary environment. The generation of connections and conduction delays is separated from the generation of neural properties to allow users to work with the simulation environment that meets their demands. After connections in Blender are defined on the level of layers using PAM, connections and distances between neurons can be computed based on given number of neurons and synapses per layer and the projection kernels for axons and dendrites. As a proof-of-concept, we implemented an importer for the neural network simulator NEST to run neural network simulations based on networks generated by PAM (see Results).

### Implementation of the hippocampal model

In the following, we demonstrate how PAM can be used to model connectivity patterns and distances between neurons based on neuroanatomical data. Note that we make no claim that this hippocampus model is complete. In our analyses, we focus on the connections between DG and CA3, CA3 and CA3, and CA3 and CA1 to reveal that with PAM structurally important features of the anatomy can be identified and incorporated in a computational model.

The projection patterns of the connections between entorhinal cortex and the hippocampal formation are also a very good example to demonstrate the benefits of PAM (e.g., Figures 3–34 and 3–41 in Andersen et al., 2006) but we do not feel confident enough in our understanding how axonal projections from the entorhinal cortex exactly enter the hippocampal formation and how the axonal projections of CA1 and subiculum project back in 3d space. Therefore, we limit ourselves to modeling the topographic relations between entorhinal cortex and the hippocampal formation in PAM. Getting the spatial form of the axonal projections right can be accomplished in the future by adding additional intermediate layers.

#### Data

The hippocampus [including dentate gyrus (DG), CA3, CA1 and subiculum] and the entorhinal cortex (medial EC, lateral EC, perirhinal cortex) were modeled based on publicly available data. The neural layers were traced in alignment with the atlas data of the Rat Hippocampus Atlas ^4^ (Kjonigsen et al., 2011) and the 3d surface model ^5^ by Ropireddy et al. (2012). The neural layers of the hippocampal formation were first traced slice by slice in coronal sections. When three-dimensional shapes are modeled in this way, regions between the slices can become very irregular due to misalignment and deformation of the slices. Therefore, in a second step the neural layers were recreated by placing vertices and edges along the natural shape of the neural layers.

Subsequently, synaptic and intermediate layers were created to define the connections. We based the model on the following reference: for the overall picture (Andersen et al., 2006), for more detailed information (Van Strien et al., 2009) and the hippocampome-project ^6^. Additionally, the Allen Brain Atlas (Jones et al., 2009), which contains a fully annotated atlas for the mouse, was consulted to extrapolate data in cases where rat data were not available to us.

#### Demonstration of the modeling advantages

In the following, we explain how previously hard to define connectivity patterns can be generated with PAM based on anatomical data.

***Connections from entorhinal cortex to DG.*** Neurons in the superficial layers of the lateral and medial entorhinal cortex (LEC and MEC) project to DG (and to CA3 and CA1). More specifically, the lateral and caudiomedial part of the LEC/MEC network projects to the septal half of DG and two more rostral areas project to the third and fourth quarter of the dentate gyrus in the temporal half (Andersen et al., 2006). To mimic the grid like density of pyramidal cells in the entorhinal cortex (Ray et al., 2014), a Voronoi-like procedural texture was generated to define the location dependent density of neurons on this layer (Figure 5D). Of course, textures generated from tracer studies could be used here to generate this effect more accurately.

**Figure 7:**
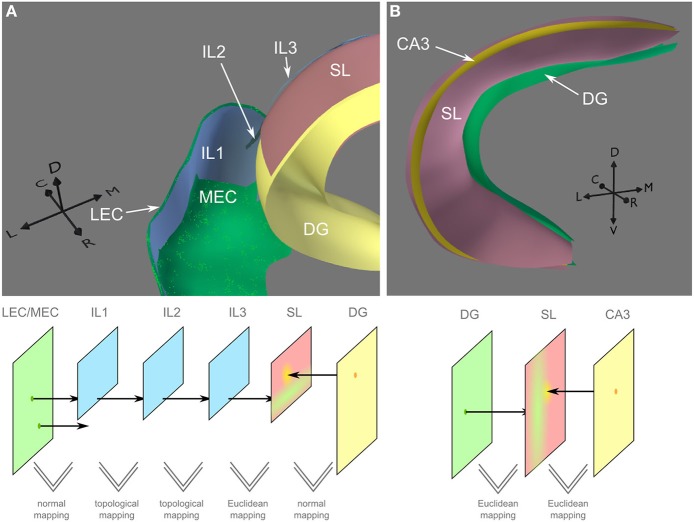
**Mapping between entorhinal cortex and dentate gyrus and between dentate gyrus and CA3. (A)** Top: Placing of neural layers (LEC/MEC, lateral/medial entorhinal cortex; DG, dentate gyrus), synaptic (SL) and intermediate layers (IL1-3). Small dots indicate placing of neurons based on texture information. Bottom: Conceptual view on the connection between LEC/MEC and DG. Colors match the 3d depiction of the top image. Using normal mapping, only a subset of LEC/MEC neurons is mapped on the first intermediate layer (IL1). Projections of those neurons pass IL2 and IL3 before they are mapped on the synaptic layer SL via Euclidean mapping. Arrows indicate lateral (L), medial (M), caudal (C), rostral (R), and dorsal (D) direction. **(B)** The synaptic layer coats CA3. Neurons in Dentate Gyrus (DG) project directly onto the synaptic layer where they can build synapses along the entire proximal-distal axis.

Intermediate layers were placed to sketch out the perforant pathway. Three subareas of the entorhinal cortex connect to different parts of the dentate gyrus. Figure 7A shows the mapping for the most caudal and lateral band to the septal portion of DG. To construct the mapping, we took advantage of the different mapping techniques in PAM. The complex relationships described in the following are also explained in a video (see http://cns.mrg1.rub.de/index.php/software). First, we created a layer (IL1) that served as a mask for the caudal lateral part of LEC/MEC, which we wanted to map to the septal portion of DG. Between LEC/MEC and IL1, we used normal mapping to ensure that only those neurons located in the caudal lateral part of LEC/MEC will project to the SL. In general, normal mapping is a helpful tool to project only a subgroup of neurons on another layer or to change the mesh structure of the layer. Additional intermediate layers (IL2 and IL3) were added to define the geometry of the perforant path. The mesh topology of IL2 and IL3 were identical to that of IL1, while their shapes were deformed to match the connection pathways of the LEC/MEC neurons. The mesh topology is used to define the relative position of the neuron projections in each layer, and the shape of the layer defines the position in 3d space. The mapping between layers IL1, IL2, and IL3 was topological since they all had the same mesh topology. From IL3, neuronal projections enter the SL using Euclidean mapping. Topological mapping could not be used here, as SL is a copy of the DG layer and, therefore, does not have the same mesh topology as IL3. Instead, using Euclidean mapping, neuronal projections on IL3, which are distributed along the septo-temporal axis, enter SL at the most posterior point of DG. From there, the connectivity kernel defines that synapses for a particular neuron can be generated along the whole proximo-distal axis of the SL (see green area on SL in Figure 7A). Normal mapping is used between SL and DG to include just the upper septal part of DG.

Note, that the spatial form of the axonal projections is only roughly sketched out in this example. In a similar manner, projections from entorhinal cortex regions to different portions of CA3 and CA1 could be modeled, but are currently not included in this model. Note, that the spatial form of the axonal projections is only roughly sketched out in this example.

***Intra-hippocampal connections.*** Granule cells in DG project to pyramidal cells in CA3, which in turn have recurrent connections and projections to CA1 cells. While connections of CA3 neurons cover nearly the entire proximal-distal axis in the hippocampal loop, their coverage along the septo-temporal axis is restricted (Ropireddy and Ascoli, 2011). Using PAM, a SL was placed between DG and CA3 (Figure 7B). As neural projections from DG should enter the SL on their shortest path and traverse along the proximal-distal axis, Euclidean mapping was used between DG and the SL. We also used Euclidean mapping between the SL and CA3, as the SL also does not have the same mesh topology as CA3 and we wanted to be sure that every CA3 neurons projects to the SL.

**Figure 8:**
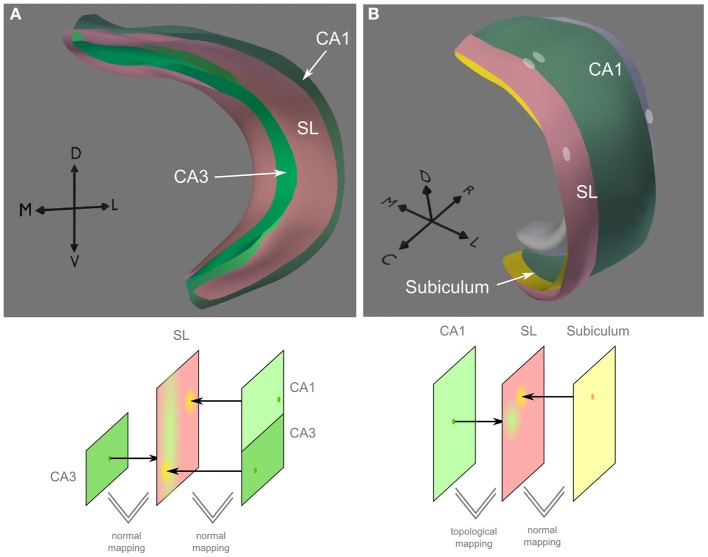
**Connections from CA3 to CA3 and to CA1 and from CA1 to subiculum. (A)** Using normal-based mapping, post-synaptic CA1 and CA3 neurons (*on the* right in lower panel) are mapped on corresponding regions of the synaptic layer. Pre-synaptic CA3 neurons project along the entire proximal-distal axis of the synaptic layer, allowing connections with CA3 and CA1 neuron. This mapping is generated in PAM in two separate steps. **(B)** The synaptic layer (SL) is topologically identical to CA1 but mirrored in the rostral-caudal axix (indicated by white dots). Thereby, neurons in the proximal part of CA1 project to distal parts of the subiculum and vice versa.

For the recurrent and forward projections of CA3, a SL covering CA3 and CA1 was created with normal-based mapping (Figure 8A). Since the SL is very close to CA3 and CA1, normal-based and Euclidean mapping yield very similar results, in particular with large connection kernels on the SL.

***Output projections.*** Pyramidal cells on the more proximal part of CA1 project to more distal parts of the subiculum and vice versa (Amaral et al., 1991). In PAM, this can easily be modeled by creating a copy of the CA1 layer, mirroring it in the caudal-rostral axis and deforming it to a SL over the subiculum (Figure 8B). As neural projections from CA1 are mapped via topological mapping on the SL, the mesh layout of the SL is used to describe the projection targets of CA1 on subiculum. Since the meshes of the SL and subiculum do not have the same topology, normal mapping is used.

**Figure 9:**
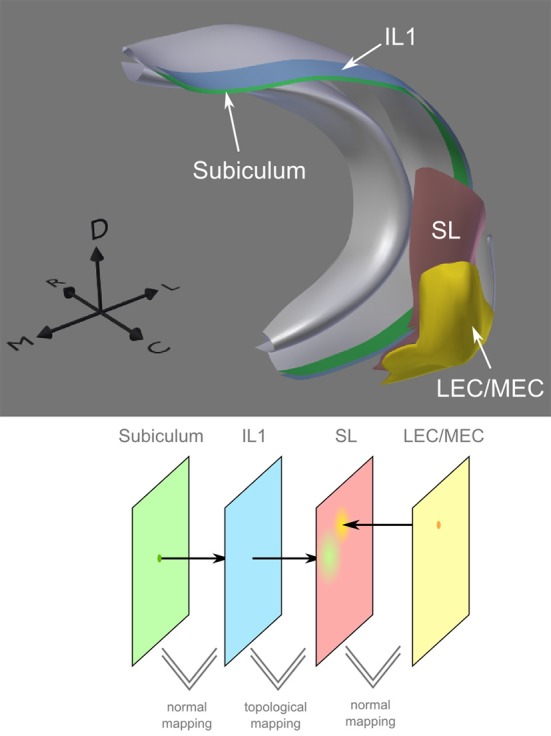
**Connections from subiculum to entorhinal cortex.** In the mapping from subiculum to entorhinal cortex, the topology remains roughly preserved (e.g., the proximal-distal axis of the subiculum maps on the medial-lateral axis of the entorhinal cortex).

CA1 and subiculum cells project back to the deep layers of LEC and MEC roughly maintaining the topological order of the cells along the septo-temporal and proximal-distal axis (Amaral et al., 1991). In PAM, this can be achieved, for instance, by normal-based mapping between the subiculum layer and an intermediate layer, which contains more subdivisions. Here, normal-based mapping is just used for a simple 1-to-1 mapping from one layer onto another with a different internal organization. A copy of this layer is deformed to match the SL close to the entorhinal cortex. Because of topologically identical shapes, projections between the intermediate layer and the SL can be directly determined. From the SL, normal-based mapping provides the link to the entorhinal layer (Figure 9).

#### Neuron and synapse numbers

In order to demonstrate that PAM can model anatomically relevant features, we will compare patterns of connectivity matrices and connection length distributions. To assess these data, it is not crucial to include realistic numbers of neurons and synapses into the model. However, the ratios of neuron numbers in different regions in our model matches experimental estimates (Amaral et al., 1990; West et al., 1991; Mulders et al., 1997; Cutsuridis et al., 2010). The total number of neurons were scaled by a factor of 0.001 (Table 1). The number of synapses per post-synaptic neuron are roughly based on experimental estimates but scaled up to allow for spike-propagation in the hippocampal loop (see last experiment).

**Table 1:** **Number of neurons and connections in the hippocampal formation used in this study**.

**From**	**Neuron numbers**	**Connectivity**				
		**EC II**	**DG**	**CA3**	**CA1**	**Sub**
sEC	110 (110,000)		38 (3520)			
DG	1200 (1,200,000)			15 (72)		
CA3	250 (250,000)			60 (6000)	85 (5500)	
CA1	390 (390,000)					15
Sub	285 (285,000)					
dEC	330 (330,000)					

The numbers are scaled based on anatomical studies in rats (Amaral et al., 1990; West et al., 1991; Mulders et al., 1997; Cutsuridis et al., 2010). Estimated neuron numbers are given in parenthesis. For the connectivity, the numbers in the columns denote the number of incoming connections that one cells receives from the regions listed in the left most column. The estimated number of incoming connections as reported in the literature (if available) is given in brackets. For example: each DG neuron in our model receives input from 38 sEC neurons. In the rat DG, it is estimated, that each neuron receives input from 3520 of sEC neurons (Cutsuridis et al., 2010).

## Results

### Qualitative view on the model

**Figure 10:**
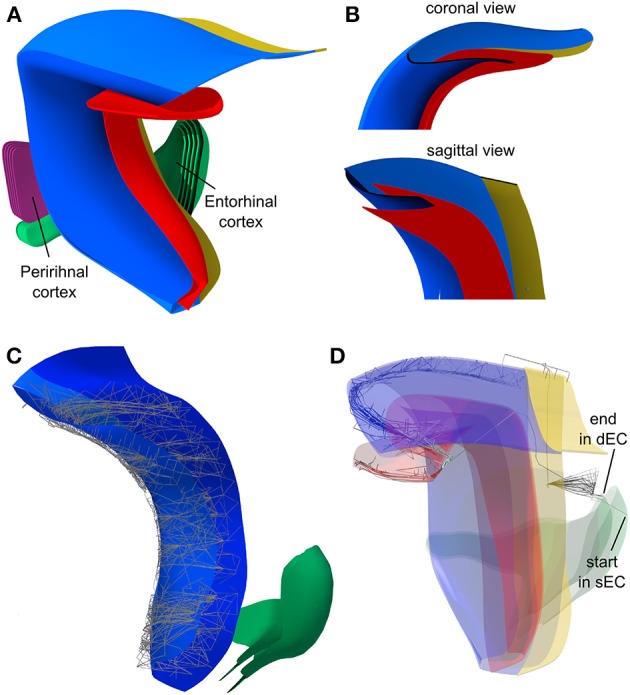
**Visualization of the reconstructed model of the hippocampus. (A)** The 3d model includes DG (red), CA3 (dark blue), CA1/CA2 (light blue), and subiculum (yellow). Furthermore, layer 6 of entorhinal cortex and perirhinal cortex were reconstructed. Layers 1–5 of these regions were added using Blender's operation “scaling along normals.” **(B)** Coronal and sagittal view on the hippocampus with clipping plane that reveals the characteristic shape of the hippocampus. **(C)** CA3-CA3 and CA3-CA1 connections along the septo-temporal axis. **(D)** Axonal projections along the hippocampal loop. Starting in superficial entorhinal cortex, projections to all post-synaptic neurons in DG are depicted. From one post-synaptic neuron on DG, all projections to the next layer are depicted, and so on, until deep entorhinal cortex is reached.

The Python implementation of PAM contains functions to visualize connections, unconnected neurons and synapse locations. Figure 10 shows the reconstructed neural layers of the hippocampal model and some visualizations of the connections computed by PAM using the intermediate layers described in the previous chapter.

### The importance of layer morphology and distance calculations

**Figure 11:**
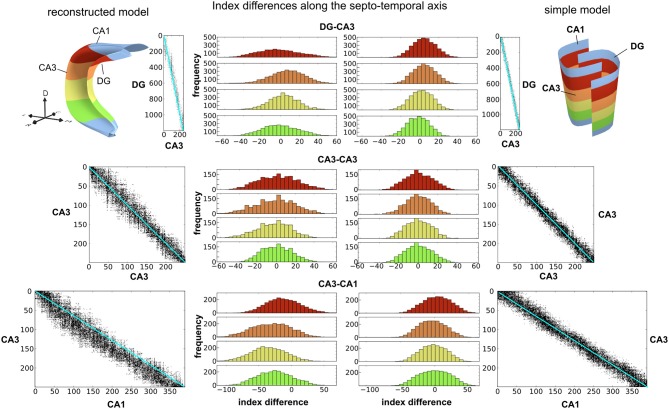
**The Morphology of the neuronal layer has a significant impact on the connectivity between neurons**. We compared the reconstructed model based on PAM (top left) to a simple model based on approximate layer morphology (top right). Results for the reconstructed and simple model are shown on the left and right, respectively. For the depiction of the connectivity matrices (scatter plots), neurons in all layers were sorted along the septo-temporal axis. Black dots in the connectivity matrices denote a connection between a pre-synaptic neuron (rows) and a post-synaptic neuron (columns). For the red, orange, yellow and green areas along the septo-temporal axis, histograms of the index differences were computed from the connectivity matrices (shown in the middle). Wider distributions indicate that the projections of a neuron are more scattered along the septo-temporal axis. Since neurons are homogeneously distributed in the layers, region sizes along the septo-temporal axis must change on the proximal-distal axis (see **Figure 12** for further explanations).

A crucial question is whether two key features of PAM, the modeling of the 3d shape of neuronal layers and the realistic calculation of connection distances, are important for the inferred connectivity patterns and distances. For illustration, we compared the connectivity and distance matrices describing DG-CA3, recurrent CA3, and CA3-CA1 connections in two models of the hippocampal formation. The reconstructed model incorporates the realistic shapes of neuronal layers in the hippocampal formation and was reconstructed in PAM from anatomical data (Figures 10, 11, left 3d model). This model is contrasted with a simple model that approximates the gross anatomical shape of the hippocampal formation as two half tubes (Figure 11, right 3d model). The simple model represents what can be generated with previously available tools such as, for example, NeuralSyns. In both models, equal numbers of neurons were homogeneously distributed over the layers and connected with equal numbers of synapses and the same connectivity kernels were used.

#### Impact on connectivity matrices

We examined how the morphology of the neural layers affected the connectivity between neurons. One way to visualize the connectivity are the connectivity matrices between two layers, where the neurons are sorted along the septo-temporal axis of the hippocampus (Figure 11, scatter plots). Both models produce connectivity matrices that are very sparse and locally restricted, as evident in the concentration of connections around the diagonal. However, in the reconstructed model, the spread along the diagonal of the connectivity matrix is wider than in the simple model. To investigate this spread in more detail, we computed the index differences between the pre-synaptic neurons and their post-synaptic targets in four regions along the septo-temporal axis (Figure 11, histograms). The index differences were calculated as the difference between the pre-synaptic index and the post-synaptic index. The connectivity spread in the reconstructed model is significantly wider than in the simple model for all anatomical subdivisions. In addition, there is another marked difference between the simple model and the reconstructed model. In the simple model, the distributions remain equal along the septo-temporal axis whereas variations are recognizable in the reconstructed model. For example, neurons in the most septal part of DG project to wider areas in CA3 than DG neurons in more temporal parts do (Figure 11, red vs. orange, yellow, or green distribution). A similar patterns of septo-temporal heterogeneity is seen for recurrent CA3 and CA3-CA1 connections.

**Figure 12:**
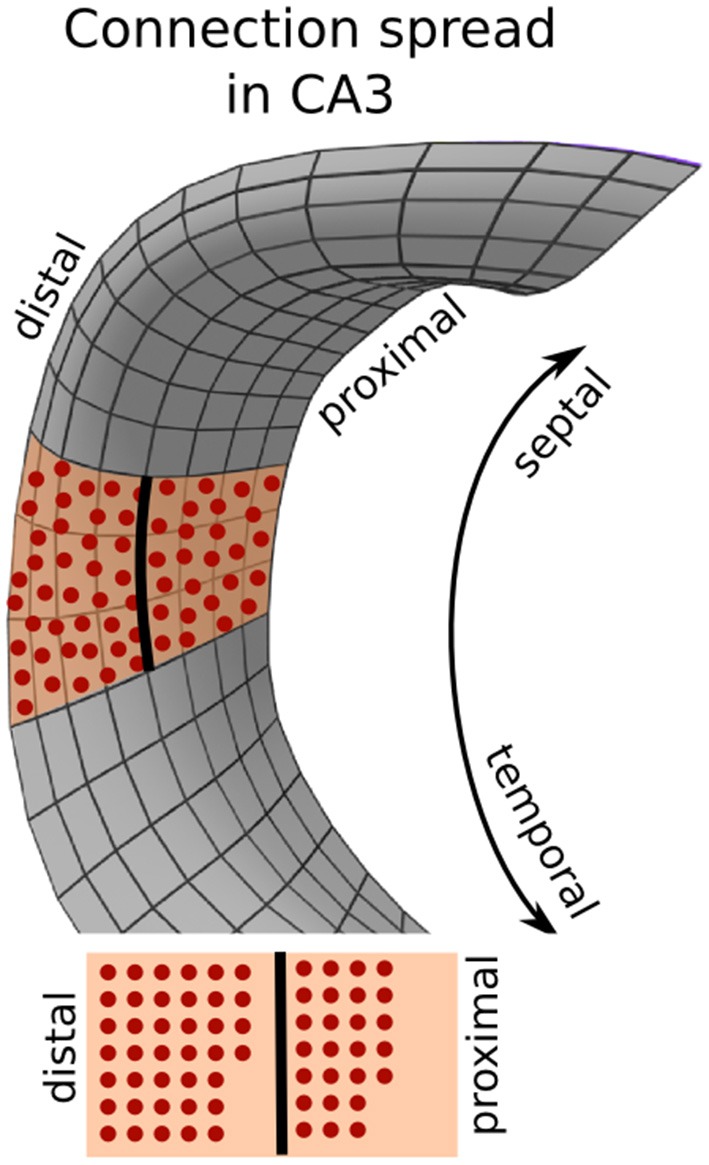
**Different surface areas between the proximal and distal parts of CA3 due to 3d morphology of the layer**. Due to the cone-like shape of the CA3 layer (the same applies for CA1), the septo-temporal axis on the proximal side is shorter than on the distal side. Given a homogeneous distribution of neurons, the distal part hosts more neurons than the proximal part.

The reason for the wider connection spread in the reconstructed model is indeed the anatomical shape of the neural layers. For example, while in the simple model, the length of CA3 at the proximal and distal part is equal, in the anatomical hippocampus, CA3 is longer at the distal end than at its proximal end. Since neurons are homogeneously distributed across the proximal-distal axis, any segment of CA3 along the septo-temporal axis must contain more neurons in the distal part than in the proximal part (Figure 12). Furthermore, since neurons in both models form the same number of connections, the projection of neurons must spread further along the septo-temporal axis in the reconstructed model than in the simple model. The other observation that the connectivity spread in the reconstructed model depends on the septo-temporal location, can be accounted for by a change in the proximal-distal asymmetry along the septo-temporal axis.

#### Significance of distance computation technique

**Figure 13:**
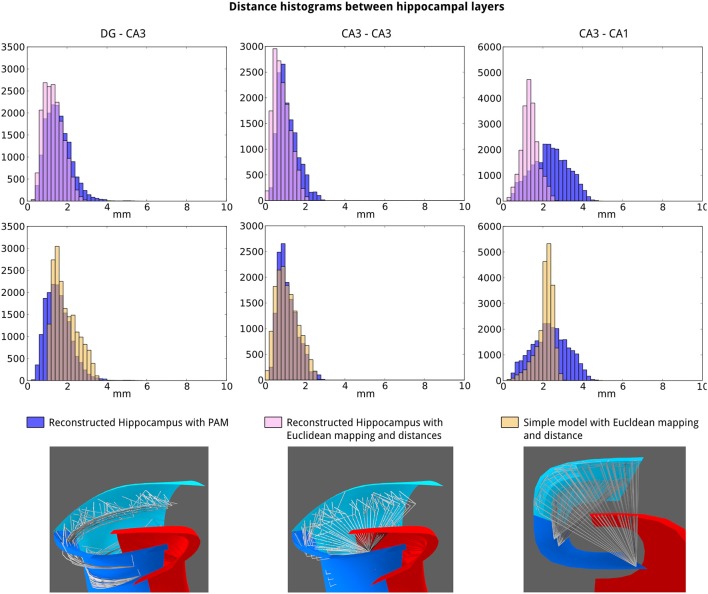
**Connection distances depend significantly on layer morphology and distance calculation model**. The distributions of connection distances for DG-CA3 **(left)**, CA3-CA3 **(middle)**, and CA3-CA1 **(right)** connections are compared for three different scenarios indicated at the lower part of the figure. The model was either the reconstructed model or the simple model. The distance calculations were either based on the PAM techniques or on the Euclidean distance.

Next, we studied how the connection distance depends on the morphology and the distance computation model in three different scenarios. In the first and second scenario, we used the reconstructed model and computed distances between neurons, respectively, based on the mapping techniques unique to PAM and based on Euclidean distance, which was available in previous tools. In the third scenario, representing the conventional approach, we used Euclidean distance in the simple model. Distance histograms were generated for DG-CA3, CA3-CA3, and CA3-CA1 connections (Figure 13).

All pair-wise comparisons between distance distributions created from PAMs and the other models using the Kolmogoroff–Smirnoff test showed significant differences (*p* < 10^−50^). These exceptionally low *p*-values are largely due to the large sample size and belies the comparatively small differences in some of the pairwise comparisons. However, for the CA3-CA1 connections, the distributions of distances are qualitatively different. The advantage of layer-based distance calculation becomes the most apparent for these connections as the axons of CA3 pyramidal cells project along the proximal-distal axis of the cornu ammonis regions rather than traversing directly to the CA1 target neurons (Figure 13, bottom).

### Effect of synaptic delays in neural network simulations

As a proof-of-concept, we imported the calculated connectivity and distance matrix from the hippocampal loop [superficial EC (sEC), DG, CA3, CA1, Sub, deep EC (dEC)] into a NEST simulation (Gewaltig and Diesmann, 2007). Due to the lack of detailed knowledge about the projections between entorhinal and hippocampal areas, connection distances represent only a rough average between the minimal and maximal spatial distances between neurons in the entorhinal cortex and dentate gyrus, CA1 and subiculum. In analogy to our previous more abstract model (Pyka and Cheng, 2014), all neurons were modeled as excitatory Izhikevich neurons (Izhikevich, 2003) (*a* = 0.02, *b* = 0.2, *r*_1_ = −65, *r*_2_ = 8) without STDP or any other sort of adaptation. Connection weights were manually adjusted to match activity levels observed in experimental studies (Leutgeb et al., 2004; Vazdarjanova and Guzowski, 2004; Tashiro et al., 2007). The weights were sEC-DG: 9 mV, DG-CA3: 5 mV, CA3-CA3: 4 mV, CA3-CA1: 5 mV, CA1-Sub: 4 mV, CA1-dEC: 4 mV, Sub-dEC: 4 mV (see also Supplemental data: hippocampus_nest/hippocampus.py).

To convert connection distances to conduction delays, the connection distances calculated in PAM were multiplied by 4.36 ms/mm according to experimental measurements of conduction latencies in rats CA3 axons (Soleng et al., 2003). Since variability and neuron-type-specific differences are not incorporated in our model, our results need to be confirmed once more information about conduction latencies and neuron-types becomes available.

**Figure 14:**
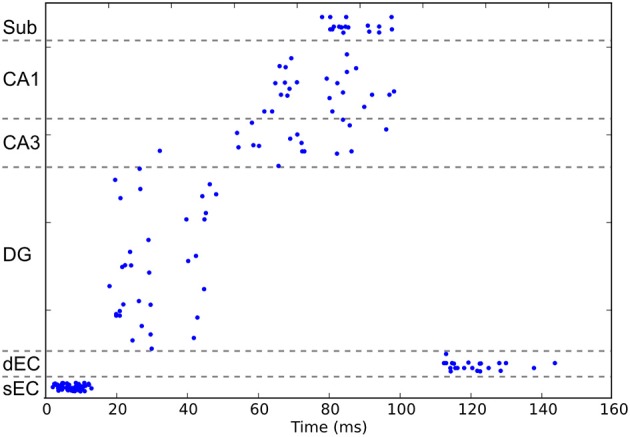
**Neural activity propagation through the hippocampal formation**. Shown are the results of network simulations with spiking neurons based on the reconstructed model and experimental evidence for distance-based conduction latencies. Coordinated spiking-activity driven by input currents to the superficial layer of entorhinal cortex propagation through the hippocampal formation and reaches the output layer, the deep layer of the entorhinal cortex, after about 120 ms. Note that EC-CA3 and EC-CA1 connections are currently not included in the model and would lead to additional spiking in the output layer during the silent period before 110 ms.

We then simulated neural activity in this network by injecting input currents created by Poisson noise into sEC for 10 ms with 50 mV. The currents are sufficient to drive spiking activity in sEC (Figure 14). After some delay these spikes in sEC in turn drive spiking activity in downstream CA3, and so on and so forth. The spiking activity finally completes the tri-synaptic loop and reaches the output layer, dEC, after around 120 ms (Figure 14), which is somewhat similar to the period of the theta oscillations at about 6–12 Hz.

## Discussion

With PAM, we introduced a technique to use anatomical data to build large scale artificial neural networks with realistic connectivity and conduction delays. In PAM, neural networks are represented by layers, which are related to each other with a set of mapping techniques. The combination of different mapping types allows us to model complex neuronal projections, e.g., between entorhinal cortex and dentate gyrus. PAM offers the unique capability to have local as well as global anatomical axes influence the connectivity patterns. Furthermore, it can combine distances between layers and within layers to calculate connection distances between neurons.

### Features of PAM and predictions

PAM is a very efficient approach to model large-scale network structures with complex wiring patterns as it mimics an important property of neural networks and biological structures in general: it indirectly describes the neural network through a lower-dimensional encoding. Spatial structures are defined for the placement and mappings of neurons and projections, rather than specifying the location and connectivity of each neuron. This low-dimensional encoding has two practical advantages. First, for the given complexity of real networks, the human effort it takes to describe position and projection directions is comparatively low. Second, the amount of data generated by the encoding is also low given the complexity of networks that can be created with PAM. Even though PAM does not include a model of the developmental process, it can be used efficiently to represent snapshots of the neural network at certain developmental stages due to the low-dimensional encoding. We believe that in combination with other properties of networks, such as neural dynamics, plasticity, and external inputs, PAM could be a valuable contribution toward a complete description of nervous systems for computational models.

We deliberately kept the computation of connections and connection distances separate from the neural network simulator, so that it is compatible with a wide range of simulation engines. Therefore, researchers who already feel comfortable with the simulator of their choice can add PAM to their workflow for generating the neural network. The connection data generated by PAM can be exported as CSV-files or as binaries using the pickle-modul of Python, which can then be imported by many other programs. An import-script for NEST is included in the downloadable package of PAM.

Based on the reconstruction of the hippocampal formation and computation of connection properties in PAM, we can derive two predictions about the structural properties of the hippocampus. First, the spread of connections is higher at the most septal locations in the hippocampus than at the more temporal locations (Figure 11). Second, conduction delays in CA3-CA1 connection have a higher variability than CA3 recurrent or DG-CA3 connections (Figure 13). Both predictions can be readily tested experimentally. Future modeling studies are needed to analyse the functional consequence of these anatomical properties.

Furthermore, we found in a preliminary simulation, that the total synaptic delay in the hippocampal formation in the current model is close to the period of the theta oscillation, which dominates the local field potential in the hippocampal formation. While the precise relationship between synaptic delays and theta oscillations needs to be ascertained in the future, we speculate at this point that there might be a correspondence between these two parameters that could account for inter-species differences in theta frequencies. As the neuron distances and, hence, the conductions delays, scale with the size of the hippocampus, we predict a relationship between brain size and the frequency of hippocampal theta, consistent with comparative studies of theta oscillations across nine species (Blumberg, 1989). This allometric relationship cannot be easily explained in models that generate theta oscillations within an isolated subregion (Crotty et al., 2012). It has to be noted that several mechanisms have been already proposed for theta and conceptually the theta frequency does not need to match the traveling time of spikes in the hippocampal loop (e.g., the frequency could be higher than suggested by the loop). However, allometric measures might constrain the range in which spike oscillations can be observed. This might be in particular relevant when brain sizes differ by several orders of magnitude.

### Morphology-based vs. kernel-based connections

For networks with spatial dependencies, it is common to use either kernel-based or morpohology-based methods to compute the connectivity and connection lengths between neurons. Kernel-based methods use two- or three-dimensional mathematical functions to define the probability for a neuron to form synapses as a function of the spatial distance to the soma of the neuron. This method provides a fast and efficient way to connect neurons with each other and is widely used in software packages like Neuroconstruct (Gleeson et al., 2007), NeuralSyns (Sousa and Aguiar, 2014), Topographica (Bednar, 2009), or NEST Topology (Eppler et al., 2008).

On the other hand, increasing effort is invested into generating networks based on realistic morphologies (Halavi et al., 2012). Tools to analyse morphologies, like the commerical software Neurolucida or Py3DN (Sousa and Aguiar, 2014) provide the data to generate artificial neurons with realistic morphology (Ascoli et al., 2001; Eberhard et al., 2006; Cuntz et al., 2011) and to generate networks, e.g., with NETMORPH (Koene et al., 2009) or NeuGen (Eberhard et al., 2006). This line of research is primarily motivated by the fact that the axonal and dendritic morphology can have a functional influence on the dynamics of the neuron (London and Häusser, 2005).

PAM combines kernel-based methods to determine synapses on a SL with structural properties of brain regions, which determine the connection patterns and their lengths. However, different types of branching morphologies of axonal and dendritic trees are not incorporated in this model. The focus of PAM lies more on an efficient translation of large-scale network morphologies to more abstract network simulations, like NEST or Brian, in contrast to GENESIS and NEURON. The unique contribution of PAM here is that topological relations between distant layers, properties along local and global anatomical axes, and anatomical data about connection pathways and cell- and synapse-densities can be modeled and converted into artificial neural networks.

However, these features do not necessarily exclude the incorporation of morphological data. In fact, we belief that approaches for generating neuron morphologies (e.g., Koene et al., 2009; Cuntz et al., 2011) could be amended by anatomical cues derived from PAM to guide the growth of dendrites and axons along layers specified in a 3d model. Thereby, a low-dimensional encoding for neuron-morphologies and network-morphologies could be generated that would allow the study of neural networks on different abstraction levels.

### Limitations of PAM and future plans

Creating PAMs requires more effort as compared to setting up more commonly used network simulations, because the 3d model has to be build from reconstructions of the anatomical data. However, we think that this effort is justified since our results show that the morphology of the neuronal layers and the mapping of connections have significant effects on connectivity and conduction delays. In addition, once the 3d model has been built, it can be used to generate an arbitrary number of network models for functional simulations due to the parametric nature of the 3d model in PAM. Parameters such as the number of neurons and synapses will have to be adjusted depending on the scientific question pursued and computational power available.

Currently, neurons and synapses are constrained to the neuronal layer, the surface of a 2d manifold in 3d space. For future versions, we plan to implement a parametric way to add a variable offset to the locations of neurons and synapses in a biologically plausible manner. Furthermore, although connections and distances can be calculated on the order of 10^5^ neurons and 10^6^ synapses in a reasonable amount of time, we have not yet exploited all possibilities to increase the computational speed. More work will be invested to allow the generation of networks with realistic numbers of neurons and synapses.

### Potential applications of PAM

We hope that PAM will help close the current gap between the computational models of neural networks, which tend to be rather abstract (Cheng, 2013) and the anatomical data, which is highly detailed. For a multitude of species, including humans, high quality structural data of the central nervous system are continuously collected and refined (e.g., Jones et al., 2009; Markram, 2012). These data proved useful in studying, for example, network properties (Soleng et al., 2003; Mason and Verwoerd, 2007; Van Strien et al., 2009), functional correlates of structural properties (Carr and Konishi, 1988; Lavenex and Amaral, 2000; Buzsáki and Moser, 2013) or genotype-phenotype relationships (Lein et al., 2007; Thompson et al., 2008). However, few neural network models are generated from structural data, possibly because an effective and powerful method to formally describe and translate those data into neural models was missing so far.

An intriguing possibility that PAM offers is to study the precise functional effect of brain lesions. Since the network models are derived from spatial anatomical data, any kind of local modification in the biological network can be reproduced in the virtual network, and vice versa. For example, controlled or known brain lesions can be simulated anatomically correctly in a virtual network. Alternatively, insights about a virtual lesion in a network simulation based on PAM can serve as prediction for *in vivo* studies. For instance, there is an ongoing debate about the functional differences between the septal and temporal part (or dorsal and ventral part in primates) of the rodent hippocampus (Thompson et al., 2008; Fanselow and Dong, 2010; Segal et al., 2010). With detailed projection patterns between neural layers, the septal and temporal part of the hippocampus can be analyzed separately in computational models generated by PAM. In general, we think that computational and experimental studies of neural networks could be more tightly integrated, if they shared a common anatomical reference frame.

The anatomical reference frame provided by PAM might prove useful in investigating the relationship between size and form of a brain structure on the one side and its impact on connectivity patterns, conduction distances, self-organizing processes and function on the other hand. This is in particular relevant to understanding the emergence of certain networks from an evolutionary point of view. For example, allometric factors can be incorporated into the analysis of networks in scaled up versions of brain structures recreated with PAM. By recreating homologous regions from different species, the functional influence of the scale and the form of a network could be dissociated with the help of computational models.

PAM could be used as a powerful educational and documentation tool. The possibility to visually explore and manipulate the reconstructed model of the hippocampus has helped us tremendously to better understand the structure of the hippocampus and the projection patterns of its neurons. In Blender, it is possible to rotate the 3d model in all three directions, remove certain layers, color layers, including making them partially transparent, and to do many more things. In addition, the Python implementation of PAM includes tools that can facilitate the understanding of the synaptic connectivity patterns. It provides functions to visualize connections and synapse locations. The description of anatomical layers and mappings between those layers as provided by PAM, could serve to collect and document knowledge about neuron locations, densities and axonal and dendritic projections.

## Conclusion

We have proposed a new modeling technique, PAM, that can generate neural networks with connectivity patterns and connection distances that are consistent with experimentally measured layer morphologies and complex projection patterns. PAM can also serve as a tool for collecting, systemizing, and visualizing anatomical data. Using a common reference frame for anatomical data would greatly facilitates the transfer of such data and increase their potential impact. It is therefore our hope that computational and experimental neuroscientists alike will find PAM a useful tool for their research.

## Information sharing statement

The Python implementation of PAM, reported in this article, along with some example files of the hippocampal model and the exported data for NEST can be found in the Supplemental Materials. PAM is further under development. The most recent version, a Wiki, and videos can be found at http://cns.mrg1.rub.de/index.php/software. We invite the reader to examine the code and contribute to the project.

## Author contributions

Martin Pyka developed the PAM technique, created the hippocampal model, Python implementation of PAM, data analyses, writing the manuscript. Sebastian Klatt Python implementation of PAM, writing the manuscript. Sen Cheng developed the PAM technique, data analyses, writing the manuscript.

### Conflict of interest statement

The authors declare that the research was conducted in the absence of any commercial or financial relationships that could be construed as a potential conflict of interest.

## Footnotes

^1^http://www.blender.org

^2^http://www.openrobots.org/wiki/morse/

^3^http://cns.mrg1.rub.de/index.php/software (will be available upon acceptance of article).

^4^http://cmbn-approd01.uio.no/zoomgen/hippocampus/home.do

^5^http://krasnow1.gmu.edu/cn3/hippocampus3d/

^6^http://www.hippocampome.org—the authors are aware, that this project is still in alpha-stadium. Therefore, data were checked in the provided reference material.
